# Associations between *HVEM/LIGHT/BTLA/CD160* polymorphisms and the occurrence of antibody-mediate rejection in renal transplant recipients

**DOI:** 10.18632/oncotarget.21941

**Published:** 2017-10-19

**Authors:** Zijie Wang, Ke Wang, Haiwei Yang, Zhijian Han, Jun Tao, Hao Chen, Yuqiu Ge, Miao Guo, Chuanjian Suo, Ji-Fu Wei, Ruoyun Tan, Min Gu

**Affiliations:** ^1^ Department of Urology, The First Affiliated Hospital With Nanjing Medical University, Nanjing, China; ^2^ School of Public Health, Nanjing Medical University, Nanjing, China; ^3^ Research Division of Clinical Pharmacology, The First Affiliated Hospital With Nanjing Medical University, Nanjing, China

**Keywords:** kidney transplantation, antibody-mediated rejection, costimulatory signals, single-nucleotide polymorphisms next generation sequencing

## Abstract

Antibody-mediated rejection (ABMR) is a serious complications that can occur following renal transplantation. The production of donor-specific antibodies by the humoral immune response can trigger costimulatory signals, which are crucial in activating immune cells, and therefore, playing a potential role in ABMR. To investigate the role of *HVEM/LIGHT/BTLA/CD160* polymorphisms in ABMR, we retrospectively analyzed 200 renal transplant recipients. We adopted next-generation sequencing (NGS) to identify *HVEM/LIGHT/BTLA/CD160* single-nucleotide polymorphisms (SNPs) in the genotypes of these patients. We divided the patients into two groups: those with ABMR and those who were stable. We adopted multiple models and performed regression analysis after adjusting for multiple confounding variables, to determine the correlation between the SNPs and ABMR. We obtained 41 high-quality SNPs readouts. However, we did not observe any significant association between these polymorphisms and the pathogenesis of ABMR in any of the models.Nevertheless, since there is evidence suggesting the involvement of costimulatory signals in graft rejection, further research should be conducted to better understand how genetic polymorphisms may be involved in ABMR.

## INTRODUCTION

Kidney transplantation is an optimal choice for patients with end-stage renal disease. It is considered superior to dialysis, due to the reduced complications, lower mortality rates and improvement to patient quality of life [1]. However, antibody-mediated rejection (ABMR), also termed as humoral rejection, poses a substantial threat to post-transplant patients and inevitably leads to allograft loss [2]. The precise pathogenesis of ABMR remains unclear. Generally, ABMR is closely associated with antibodies ligating to donor antigens, which mediate allograft damage via activation of the complement system or cytotoxic cells [3, 4]. These antibodies are directed against human leukocyte antigens (HLAs) and major histocompatibility complex (MHC) class I and II antigens, termed as donor-specific antibodies (DSAs) [5]. Meanwhile, they can also be directed against other stimulators, such as minor histocompatibility antigens, ABO group antigens and endothelia cell antigens [6, 7]. Although ABMR occurs in less than 10% of renal transplant recipients, 30% of them ultimately suffer from graft loss [3]. As a result, ABMR impacts the long-term graft survival in kidney transplantation and is one of the most challenging clinical events following renal transplant [8].

Activation of both T and B cells after transplantation is a tightly regulated process consisting of multiple distinct but interrelated signals [9]. Secondary signals, also named costimulatory signals, play an important role in activation and inhibition of immune cells. Recently, much attention has been placed on HVEM (herpes virus entry mediator) and LIGHT (homologous to lymphotoxin, which exhibit inducible expression and compete with HSV glycoprotein D for binding to HVEM, a receptor expressed on T lymphocytes), BTLA (B and T lymphocyte attenuator) and CD160 costimulatory pathways. HVEM and LIGHT belong to the TNFR superfamily, while BTLA and CD160 are members of the Ig superfamily. The functions and structures of these costimulatory molecules can be divided into positive and negative costimulatory pathways [10]. The binding of HVEM on T cells to membrane-bound LIGHT delivers positive signals through HVEM that promotes T-cell survival, while the conjugation of HVEM to CD160/BTLA on T cells delivers a coinhibitory signal that deactivates T-cells [11–14]. There is substantial evidence that suggests disorder of the HVEM/LIGHT/BTLA/CD160 signaling system is essential in the development of autoimmune diseases and allograft rejection [15, 16]. Costimulatory signals are widely investigated in T cell mediated immunity. However, regarding humoral immunity, the role of the HVEM/LIGHT/BTLA/CD160 costimulatory system in B cell activation and allograft transplantation remains unclear. Studies suggest that HVEM is expressed at high levels in all peripheral blood B cells, while at low levels in germinal center (GC) B lymphocytes, which may be activated since GC is where dendritic cells (DC), T cells and B cells interact [17]. It is postulated that LIGHT expression on DC and T cells causes HVEM engagement on naïve B cells, which costimulates B cell proliferation and Ig secretion, as a result, enhancing humoral immune responses [13]. It has also been suggested that *de novo* DSAs is needed in the cognate interaction between CD4^+^ T follicular helper cells (Tfh), which are primed by donor alloantigens and presented as host antigen presenting cells and B lymphocytes that recognize soluble and membrane-bound alloantigens. This suggests the possibility that HVEM/LIGHT/BTLA/CD160 participates in the modulation of DSAs and humoral immune response [18–20].

Until now, the association between *HVEM/LIGHT/BTLA/CD160* gene polymorphisms and ABMR in renal transplant recipients has remained unexplored. Here, we evaluated the association between a total 41 single nucleotide polymorphisms (SNPs) of *HVEM/LIGHT/BTLA/CD160* genes and occurrence of ABMR and investigated its role in the formation of DSAs and pathogenesis of ABMR in renal transplantation recipients.

## RESULTS

### Demographic and clinical characteristics

The demographic characteristics of the renal transplant recipients are shown in Table 1. This study included 200 patients from the Chinese Han population: 69 renal transplant recipients had ABMR (40 men and 29 women), while 131 were considered stable (82 men and 49 women). The immunosuppressive protocols administered in stable and ABMR groups are also presented. Among patients in ABMR groups, we further collected ABMR-related clinical information, such as C4d scoring, histological classifications and the level of serum DSAs, and reported them in Table 1. We did not observe any significant differences (*P*>0.05) in age, sex, donor type and immunosuppressive protocol between the stable and ABMR group.

**Table 1:** Basic characteristics of patients included in our study

Characteristics	Stable group	ABMR group	*P* value
**Case number**	131	69	NS
**Age (years; mean ± SD)**	38.56 ± 1.40	38.92 ± 1.02	NS
**Male (%)**	62.60	57.97	NS
**PRA (%)**	0	0	NS
***Donor type***			NS
**Living-related**	16	7	
**DCD**	115	62	
***Immunosuppressive protocol***			NS
**Pred + MMF + CsA**	62	26	
**Pred + MMF + TAC**	60	35	
**Pred + MMF + CsA + SIR**	5	6	
**Pred + MMF + TAC + SIR**	4	2	
Type of ABMR*			
**Acute ABMR**	-	23	
**Chronic active ABMR**	-	46	
Grade of morphologic tissue injury^*^			
**Grade I**	-	25	
**Grade II**	-	34	
**Grade III**	-	10	
C4d Scroing by IF^*^			
**C4d1**	-	5	
**C4d2**	-	17	
**C4d3**	-	47	
***Criculating DSAs (MFI, mean ± SD)***			
**Class I**	-	1368.12 ± 550.96	
**Class II**	-	1191.23 ± 655.88	

**Abbreviations:** ABMR, antibody-mediated rejection; NS, not significant; SD, standard deviation; PRA, panel reactive antibody; Pred, prednisone; MMF, Mycophenolate Mofetil; CsA, Cyclosporin A; TAC, tacrolimus; SIR, sirolimus; IF, immunofluorescence; DSA, donor-specific antibody.

^*^The classification of ABMR are in accordance with Banff 2007 criteria.

### Association of *HVEM/ LIGHT/ BTLA/ CD160* SNPs with ABMR

Previous investigations into *HVEM/LIGHT/BTLA* SNPs have been limited to rs2234163, rs2234165 and rs2234167 for *HVEM* SNPs, rs344560 and rs2291667 for *LIGHT* SNPs, and rs9288952, rs2171513 and rs76844316 for *BTLA* SNPs. However, in our study, we screened the genetic distribution of 41 *HVEM*/*LIGHT/BTLA*/*CD160* SNPs, which we show in Table 2. All genotype frequencies in the control group conformed to the Hardy-Weinberg equilibrium (HWE) (*P*>0.05; Table 2). In logistic regression analysis and corrected for age, sex, and immunosuppressive protocols (Table 3), we did not find any significant associations (*P*<0.05) between the occurrence of ABMR and polymorphisms in any of the 41 *HVEM/LIGHT/BTLA/CD160* SNPs among the different models.

**Table 2:** Genetic distributions of *HVEM/LIGHT/BTLA/CD160* polymorphisms between the ABMR and stable group

Genotype	Chromosome	Position	Stable group (n=131)	ABMR group (n=69)	HWE for the stable group	
					Χ2	*P* value
**HVEM**						
rs4870	Chr1	2488153			0.74	0.69
AA			38	24		
AG			70	34		
GG			23	11		
rs2234158	Chr1	2489200			<0.01	0.99
CC			103	69		
CT			1	0		
rs376994775	Chr1	2489746			<0.01	0.99
CC			130	69		
CT			1	0		
rs754021885	Chr1	2489961			<0.01	0.99
CC			130	68		
CT			1	1		
rs572222644	Chr1	2491163			<0.01	0.99
CC			131	68		
CT			0	1		
rs2234161	Chr1	2491205			<0.01	0.99
CC			36	20		
CT			65	35		
TT			30	14		
rs2234162	Chr1	2491305			<0.01	0.99
CC			130	69		
CT			1	0		
rs2234163	Chr1	2491306			0.23	0.89
GG			123	64		
GA			8	5		
rs2234165	Chr1	2492276			0.39	0.82
GG			120	63		
GA			11	6		
rs575127151	Chr1	2492935			<0.01	0.99
GG			131	68		
GA			0	1		
rs375010878	Chr1	2493087			<0.01	0.99
CC			130	68		
CT			1	1		
rs2234167	Chr1	2494330			0.16	0.92
GG			123	66		
GA			8	3		
rs8725	Chr1	2494785			0.01	0.99
GG			37	19		
GA			64	35		
AA			30	15		
rs376495994	Chr1	2496492			<0.01	0.99
GG			131	68		
GA			0	1		
rs186536172	Chr1	2496521			<0.01	0.99
CC			130	68		
CT			1	1		
rs7544646	Chr1	2496649			0.38	0.83
CC			47	19		
CG			59	35		
GG			25	15		
rs7515633	Chr1	2496653			0.18	0.91
AA			43	19		
AG			61	35		
GG			27	15		
						
**LIGHT**						
rs344560	Chr19	6665020			0.55	0.76
TC			13	7		
CC			118	62		
rs772372888	Chr19	6665098			<0.01	0.99
CC			130	69		
CT			1	0		
rs61761328	Chr19	6665099			0.02	0.99
GG			127	69		
GA			4	0		
rs183886666	Chr19	6665336			<0.01	0.99
GG			131	68		
GA			0	1		
rs8101047	Chr19	6665481			1.24	0.54
AA			4	0		
AG			38	25		
GG			89	44		
rs542346038	Chr19	6667076			<0.01	0.99
GG			130	69		
GA			1	0		
rs2291668	Chr19	6669934			4.37	0.11
GG			63	26		
GA			57	38		
AA			11	5		
rs2291667	Chr19	6669986			0.05	0.98
GG			128	66		
GA			3	3		
rs748673655	Chr19	6669992			<0.01	0.99
CC			130	69		
CT			1	0		
rs344558	Chr19	6670253			0.04	0.98
AA			114	56		
AC			17	12		
CC			0	1		
rs563748272	Chr19	6677752			<0.01	0.99
GG			130	69		
GT			1	0		
						
**BTLA**						
rs2971205	Chr3	112184772			<0.01	0.99
AA			130	69		
AG			1	0		
rs2171513	Chr3	112184927			3.07	0.22
AA			15	3		
AG			42	25		
GG			74	41		
rs770019001	Chr3	112184932			<0.01	0.99
CC			130	69		
CG			1	0		
rs9288952	Chr3	112185025			3.87	0.14
GG			24	5		
GA			48	29		
AA			59	35		
rs76844316	Chr3	112188609			0.02	0.99
TT			110	61		
TG			20	8		
GG			1	0		
rs16859629	Chr3	112190380			<0.01	0.99
TT			130	69		
TC			1	0		
rs9851198	Chr3	117448419			<0.01	0.99
GG			131	68		
AA			0	1		
						
**CD160**						
rs2231375	Chr1	145696694			0.67	0.71
GG			96	55		
GA			31	13		
AA			4	1		
rs3766526	Chr1	145698637			<0.01	0.99
GG			131	68		
GA			0	1		
rs368476773	Chr1	145698914			<0.01	0.99
CC			131	68		
CT			0	1		
rs193141418	Chr1	145698935			0.06	0.97
CC			125	68		
CT			6	1		
rs587741068	Chr1	145703913			<0.01	0.99
AA			130	69		
AG			1	0		
rs587727931	Chr1	145704474			<0.01	0.99
GG			130	69		
GA			1	0		

**Abbreviations:** ABMR, antibody-mediated rejection; HVEM, herpes virus entry mediator; LIGHT, homologous to lymphotoxin (lymphotoxin-like), exhibits inducible expression and competes with HSV glycoprotein D for binding to herpesvirus entry mediator, a receptor expressed on T lymphocytes; BTLA, B and T lymphocyte attenuator; CD, cluster of differentiation; NA, not available; HWE, hardy-weinberg equilibrium.

**Table 3:** Regression analysis for age-, sex- and immunosuppressive protocol-adjusted *BTLA/HVEM/CD160/LIGHT* genetic polymorphisms among recipients with ABMR

SNPs	model	OR	95%CIs	*P* value
**rs2971205**				
	Additive	NA	NA	1.00
	Dominant	NA	NA	1.00
**rs2171513**				
	Additive	0.81	0.51, 1.29	0.38
	Dominant	0.92	0.50, 1.69	0.79
	Recessive	0.36	0.10, 1.33	0.13
	HET	1.11	0.59, 2.11	0.74
	HOM	0.38	0.10, 1.41	0.15
**rs770019001**				
	Additive	NA	NA	1.00
	Dominant	NA	NA	1.00
**rs9288952**				
	Additive	0.74	0.48, 1.15	0.18
	Dominant	0.85	0.47, 1.55	0.60
	Recessive	0.37	0.13, 1.03	0.06
	HET	1.08	0.57, 2.04	0.82
	HOM	0.38	0.13, 1.11	0.08
**rs76844316**				
	Additive	0.72	0.30, 1.70	0.45
	Dominant	0.74	0.30, 1.81	0.51
	Recessive	NA	NA	1.00
	HET	0.77	0.31, 1.89	0.57
	HOM	NA	NA	1.00
**rs16859629**				
	Additive	NA	NA	1.00
	Dominant	NA	NA	1.00
**rs9851198**				
	Additive	NA	NA	1.00
	Dominant	NA	NA	1.00
**rs4870**				
	Additive	0.83	0.53, 1.28	0.39
	Dominant	0.77	0.41, 1.45	0.42
	Recessive	0.80	0.36, 1.78	0.58
	HET	0.80	0.41, 1.56	0.50
	HOM	0.69	0.28, 1.70	0.42
**rs2234158**				
	Additive	NA	NA	1.00
	Dominant	NA	NA	1.00
**rs376994775**				
	Additive	NA	NA	1.00
	Dominant	NA	NA	1.00
**rs754021885**				
	Additive	3.06	0.18, 52.98	0.44
	Dominant	3.06	0.18, 52.98	0.44
**rs572222644**				
	Additive	NA	NA	1.00
	Dominant	NA	NA	1.00
**rs2234161**				
	Additive	0.92	0.60, 1.40	0.68
	Dominant	0.96	0.49, 1.86	0.90
	Recessive	0.81	0.39, 1.68	0.58
	HET	1.02	0.51, 2.06	0.95
	HOM	0.82	0.35, 1.94	0.66
**rs2234162**				
	Additive	NA	NA	1.00
	Dominant	NA	NA	1.00
**rs2234163**				
	Additive	1.83	0.66, 5.04	0.24
	Dominant	1.66	0.54, 5.11	0.38
**rs2234165**				
	Additive	1.01	0.35, 2.91	0.99
	Dominant	1.01	0.35, 2.91	0.99
**rs575127151**				
	Additive	NA	NA	1.00
	Dominant	NA	NA	1.00
**rs375010878**				
	Additive	2.39	0.14, 40.69	0.55
	Dominant	2.39	0.14, 40.69	0.55
**rs2234167**				
	Additive	0.80	0.20, 3.17	0.75
	Dominant	0.80	0.20, 3.17	0.75
**rs8725**				
	Additive	0.98	0.65, 1.50	0.94
	Dominant	1.05	0.54, 2.05	0.88
	Recessive	0.90	0.44, 1.83	0.76
	HET	1.10	0.54, 2.23	0.79
	HOM	0.95	0.41, 2.22	0.91
**rs376495994**				
	Additive	NA	NA	1.00
	Dominant	NA	NA	1.00
**rs186536172**				
	Additive	2.28	0.13, 39.34	0.57
	Dominant	2.28	0.13, 39.34	0.57
**rs7544646**				
	Additive	1.22	0.81, 1.85	0.34
	Dominant	1.48	0.77, 2.84	0.24
	Recessive	1.12	0.54, 2.34	0.76
	HET	1.50	0.75, 3.13	0.23
	HOM	1.43	0.61, 3.35	0.41
**rs7515633**				
	Additive	1.12	0.74, 1.69	0.60
	Dominant	1.29	0.67, 2.49	0.44
	Recessive	1.02	0.49, 2.11	0.96
	HET	1.33	0.66, 2.68	0.42
	HOM	1.21	0.52, 2.83	0.66
**rs2231375**				
	Additive	0.71	0.37, 1.34	0.29
	Dominant	0.68	0.33, 1.40	0.29
	Recessive	0.59	0.06, 5.47	0.64
	HET	0.70	0.33, 1.46	0.34
	HOM	0.54	0.06, 5.09	0.59
**rs3766526**				
	Additive	NA	NA	1.00
	Dominant	NA	NA	1.00
**rs368476773**				
	Additive	NA	NA	1.00
	Dominant	NA	NA	1.00
**rs193141418**				
	Additive	0.27	0.03, 2.35	0.24
	Dominant	0.27	0.03, 2.35	0.24
**rs587741068**				
	Additive	NA	NA	1.00
	Dominant	NA	NA	1.00
**rs587727931**				
	Additive	NA	NA	1.00
	Dominant	NA	NA	1.00
**rs344560**				
	Additive	1.01	0.38, 2.69	0.99
	Dominant	1.01	0.38, 2.69	0.99
**rs772372888**				
	Additive	NA	NA	1.00
	Dominant	NA	NA	1.00
**rs61761328**				
	Additive	NA	NA	1.00
	Dominant	NA	NA	1.00
**rs183886666**				
	Additive	NA	NA	1.00
	Dominant	NA	NA	1.00
**rs8101047**				
	Additive	1.02	0.58, 1.81	0.94
	Dominant	1.18	0.63, 2.21	0.60
	Recessive	NA	NA	1.00
	HET	1.31	0.70, 2.47	0.40
	HOM	NA	NA	1.00
**rs542346038**				
	Additive	NA	NA	1.00
	Dominant	NA	NA	1.00
**rs2291668**				
	Additive	1.20	0.74, 1.94	0.46
	Dominant	1.51	0.82, 2.79	0.18
	Recessive	0.66	0.21, 2.07	0.48
	HET	1.65	0.88, 3.08	0.12
	HOM	0.87	0.26, 2.88	0.82
**rs2291667**				
	Additive	2.32	0.43, 12.64	0.33
	Dominant	2.32	0.43, 12.64	0.33
**rs748673655**				
	Additive	NA	NA	1.00
	Dominant	NA	NA	1.00
**rs344558**				
	Additive	1.77	0.80, 3.95	0.16
	Dominant	1.67	0.72, 3.89	0.23
	Recessive	NA	NA	1.00
	HET	1.53	0.65, 3.62	0.33
	HOM	NA	NA	1.00
**rs563748272**				
	Additive	NA	NA	1.00
	Dominant	NA	NA	1.00

Abbreviations: SNPs, single nuclear polymorphisms; OR, odds ratio; CIs: confidential intervals.

## DISCUSSION

To the best of our knowledge, this is the first study that deploys next-generation sequencing (NGS) technology to investigate the association between *HVEM/LIGHT/BLTA/CD160* SNPs and ABMR in renal transplant recipients. We screened a total 41 SNPs, previously unexplored in the context of ABMR, and show that none of the polymorphisms were significantly associated with the onset of ABMR in renal transplant recipients.

HVEM belongs to the TNF receptor superfamily and acts as a shared ligand for the costimulatory and coinhibitory receptor [13]. Human HVEM is a type 1 transmembrane glycoprotein with four pseudo repeats of the cysteine-rich domain (CRD) in its extracellular domain. It is expressed widely on T cells, B cells and other hematopoietic (DC, Tregs, monocytes, neutrophils, and NK cells) and nonhematopoietic cells (parenchymal cells) [13]. HVEM serves a central role in the *HVEM/LIGHT/BTLA/CD160* costimulatory pathway, directing both positive (LIGHT) and negative (BTLA/CD160) costimulatory signals depending its receptor [21]. Rs2234163, rs2234165 and rs2234167, which are included in our study, have been researched in association with *HVEM* polymorphism and sporadic breast cancer [22]. In this instance, Dalin Li et al. reported that rs2234167, which is in the exon of the *HVEM* gene, is significantly associated with increased breast cancer risk, and presumed to influence the binding affinity between *HVEM* and *BTLA/LIGHT/CD160* [22]. In our study, however, we did not find any significant association between 17 SNPs of *HVEM* and the onset of ABMR in renal transplant recipients.

LIGHT, a member of the TNF cytokine superfamily, is a type II transmembrane glycoprotein that is widely expressed on hematopoietic cells at certain periods of cell differentiation, including T cells, B cells, DC, NK cells and platelets, acting as a key cytokine with multiple functions [13, 23]. LIGHT-deficient mice survived slightly longer than control mice (10 days versus 7 days) in fully MHC-mismatched cardiac transplantation, implying that the *HVEM/LIGHT* pathway has potential functions in transplantation [24]. Meanwhile, in the humoral immune response, recent work suggests that LIGHT participates in B cell expansion and promotes Ig production [17]. LIGHT binds to three receptors: HVEM, LTβR and DcR3. The human LIGHT gene is situated on a segment of chromosome 19p13.3, which is paralogous to the MHC immune response loci [23]. Previous investigations have demonstrated that rs344560, located near the receptor-binding region of LIGHT, directly influences the binding avidity to LTβR, whereas rs2291667, positioned in the cytosolic domain, which could decrease the binding avidity to DcR3 and lowers the expression of *LIGHT* on the cell membrane [25]. Heterotrimers of SNPs are associated with lower DcR3 avidity and the increased *LIGHT* bioavailability, contributing to the pathogenesis of inflammatory diseases, such as rheumatoid arthritis. However, in our study, we did not find any significant differences in SNP distributions on *LIGHT* genes between the ABMR and control group of renal transplant recipients, calling for a deeper investigation into the functions of *LIGHT* in ABMR.

The *BTLA* gene is located on chromosome 3 in q13.2 with five exons [26]. BTLA is a member of the immunoglobulin superfamily and is constitutively expressed on naïve T and B cells, NK cells, macrophages and dendritic cells at low levels [10]. BTLA is up-regulated on activated T cells, but when conjugated with HVEM, a co-inhibitory signal suppresses T cell activation and differentiation *in vitro* [12]. Studies regarding the genetic variations of *BTLA* have mainly focused on its role in cancer (for example, lymphocytic leukemia [27] and breast cancer [28]) and susceptibility to autoimmune diseases (for example, rheumatoid arthritis [29, 30], systemic lupus erythematosus and type 1 diabetes mellitus [31]). In particular, the majority of investigation have focused on rs9288952 and its role in increasing breast cancer risk in Chinese populations [28] and rheumatoid arthritis in Japanese and Taiwanese populations [30, 31]. Inuo et al. revealed no relationship between rs2171513 and susceptibility to lupus erythematosus and type 1 diabetes mellitus in Japanese populations [31]. While, Oki et al. showed rs76844316 is significantly related to rheumatoid arthritis in Japanese populations [29]. Our study is the first to investigate the association between *BTLA* SNPs with the development of ABMR in renal transplant recipients. None of the seven *BTLA* SNPs we screened, including the three SNPs mentioned above, showed any association with ABMR.

CD160 is another member of the Ig superfamily and is glycosylphosphatidylinositol anchored on the cell membrane [32]. It is also the second co-inhibitory ligand of HVEM, commonly associated with cytolytic activity in NK, NKT, and CD8+ T cells [33]. A recent study suggests that CD160 signaling is vital in activating CD28-independent effector/memory CD8+ alloreactive T cells. This is because CD160Ig inhibits alloreactive CD8+ T cell proliferation and IFN-γ production *in vitro*, particularly in the absence of CD28 costimulation, resulting in the prolonged survival of fully mismatched cardiac allograft in CD4-/-, CD28-/- knockout and CTLA4Ig treated wild type recipients [34]. However, there are no studies available that address the association between CD160 and humoral immunity, including polymorphism of CD160 and the onset of allograft rejection. In our study, none of the six CD160 SNPs showed any significant association with the occurrence of ABMR in renal transplant recipients.

This study is a first attempt in addressing the functions of *HVEM/LIGHT/BTLA/CD160* cosignaling pathway in the pathogenesis of ABMR in renal transplant patients. While our results suggest that the 41 *HVEM/LIGHT/BTLA/CD160* SNPs that we screened are not associated with the onset of ABMR, there are several advantages in our approach. First, we collected sufficient baseline information about the patients and included an adequate number of control patients. Second, we adopted NGS technology, which allows high-throughput and large-scale analysis of the genotypes, increasing the reliability of our findings. Third, we used regression analysis after adjusting the data for multiple confounding factors to obtain more detailed clinical information. Moreover, considering various causing contribute to the pathogenesis of post-transplant ABMR, we failed to collect more ABMR-related information to analysis the distributions of these causing and its relationship with SNPs in our study. Therefore, further studies are required with larger sample sizes from different populations to fully understand the role of these genes in ABMR onset.

In summary, through a case-control study on 69 renal transplant recipients with ABMR and 131 control recipients, we provide the first study to explore the association between *HVEM/LIGHT/BTLA/CD160* gene polymorphisms and ABMR in renal transplant recipients. We showed that none of the 41 *HVEM/LIGHT/BTLA/CD160* gene polymorphisms were associated with ABMR. Since there are limited studies investigating the role of the costimulatory signaling pathways in graft rejection, we recommend further research is required to gain a deeper understanding of the role of these genes and its variants in ABMR after kidney transplantation.

## MATERIALS AND METHODS

### Ethics statement

The procedures followed in our study were in accordance with the ethical standards of the Declarations of Helsinki and Istanbul. The study was limited to the living-related transplantation of kidney tissues to lineal or collateral relatives not beyond the third degree of kinship, or transplantation of kidney tissues from cadaveric allograft donors after cardiac death. The protocols followed were approved by the local ethics committee of The First Affiliated Hospital with Nanjing Medical University. We obtained written informed consent from all transplant recipients. None of the transplant donors were considered vulnerable.

### Collection of patient data

The study included 200 renal transplant recipients who underwent kidney transplantation between February 2008 and December 2015 at the Kidney Transplant Center of The First Affiliated Hospital of Nanjing Medical University. At least two clinicians critically reviewed the transplant recipients’ medical records, and extracted relevant data, including age, gender, transplant date, duration of transplantation, number of transplants, and immunosuppressive protocol, for patient selection. They also extracted data on panel reactive antibodies and HLA mismatch during the pre-transplant period.

Methylprednisolone was intravenously administered at a dosage of 500 mg/day during surgery and for two days following the procedure. Following this, the dosage was reduced to 400 mg, 300 mg, 200 mg, and then 80 mg on each subsequent day. This was followed by administration of prednisone at a dosage of 30 mg/day as maintenance therapy. In addition, basiliximab (20 mg) was intravenously administered 30 min before the procedure and on the fourth day after the procedure. All recipients received a three-drug or four-drug immunosuppressive regimen: cyclosporin A (n = 101) or tacrolimus (n = 99) combined with mycophenolate mofetil and prednisone, with or without sirolimus (n = 17). The starting dose of cyclosporine A and tacrolimus was 8 mg·kg^-1^·day^-1^ and 0.2 mg·kg^-1^·day^-1^, respectively; these doses were later adjusted according to serum creatinine levels. In patients where ABMR episodes occurred, methylprednisolone was intravenously administered at a dosage of 200 mg/day for three to five days.

### Diagnosis of antibody-mediated rejection

We considered an increase in serum creatinine by 20% from the baseline (not attributable to other causes), fever, proteinuria and pain in the region of the transplanted kidney to be indicative of ABMR. To confirm the diagnosis, we analyzed allograft biopsies according to the Banff 07 classification criteria, which included positive C4d staining, presence of circulating DSAs and morphological evidence of acute tissue injury [35]. Moreover, patients diagnosed with either acute ABMR or chronic active ABMR were all included in our study.

### Sample collection, preparation and NGS

We collected peripheral blood samples (2 mL) from each recipient and extracted DNA using the QIAmp DNA mini kit (Qiagen, Hilden, Germany). We quantitatively analyzed the concentration and purity of genomic DNA (gDNA) using NanoDrop ND2000 (Thermo, MA, USA), and assessed gene integrity using agarose gel electrophoresis. We considered gDNA samples with a total mass of ≥1 μg and A260/A280 absorbance ratio of ≥1.80 and ≤2.0 as acceptable. Then, we selected a pool containing upstream and downstream oligonucleotides specific to the target regions of interest as gDNA hybrids. We next fragmented gDNA using a Bioruptor Interrupt instrument (Diagenode, Belgium), and performed quantitative detection to ensure that the average fragment size was 150–250 bp. We then performed end repair, dA-tailing and sequencing adaptor ligation using the ABI 9700 PCR instrument (ABI, USA). We amplified the adapter-ligated DNA by selective, limited-cycle PCR for five cycles, before quantitatively analyzing using the Qubit dsDNA HS Assay Kit (Invitrogen, USA). We hybridized the prepared library (750 ng) with 11 μL of hybridization blocking buffer (Allwegene, China), 20 μL of hybridization buffer (Allwegene, China) and a mixture of 5 μL RNase block (Invitrogen, USA) and 2 μL probes (Allwegene, China) overnight (at least 8–16 h) at 65°C. We mixed the hybridized products with 200 μL Dynabeads MyOne Streptavidin T1 magnetic beads (Invitrogen, USA) for 30 min at room temperature. The products were then washed twice with a wash buffer (Allwegene, China), before the mixture was amplified for 16 PCR cycles and quantitatively assessed using the Qubit dsDNA HS Assay Kit (Invitrogen, USA). We denatured the captured libraries and loaded them onto an Illumina cBot instrument at a concentration of 12 to 16 pmol/L for cluster generation, according to the manufacturer’s instructions. We sequenced up to 20 WUCaMP libraries per HiSeq lane. A PhiX control (Illumina) was added to lane 8 of each flow cell.

### Analysis of NGS data

We analyzed sequencing data, including the number of altered chromosomes, genomic alterations and the depth of the sequencing coverage. We based all analyses on the human reference sequence UCSC hg19 assembly (NCBI build 37.2) using the Burrows-Wheeler Aligner. We performed local alignment and duplication removal using the Genome Analysis Tool Kit and Picard software. We detected SNPs using dbSNP 132. We used Gemini software to detect damaging or deleterious SNPs and prediction tools such as Sorting Intolerant from Tolerant and Polymorphism Phenotyping to analyze all human non-synonymous SNPs. In addition, we detected putative somatic variant calls with two separate programs: MuTect 1.1.5 and VarScan 2.3.6, by pairing each sample with its matched blood sample.

### Statistical analysis

We determined conformance to the HWE using genotype frequencies obtained from a single gene. We used the chi-square test to compare the observed and expected values. We performed genotype association analysis using a dominant model (minor allele homozygotes plus heterozygotes vs. major allele homozygotes), recessive model (minor allele homozygotes vs. heterozygotes plus major homozygotes), additive model (major homozygotes vs. heterozygotes vs. minor homozygotes), HET model (major homozygotes vs. heterozygotes) and HOM model (major homozygotes vs. minor homozygotes). We compared genotypic frequencies between the control and ABMR group using the chi-square test. In addition, we explored linkage disequilibrium blocks using Haploview version 4.2. We calculated odds ratios (ORs) and 95% confidence intervals (95% CIs) using the SPSS 13.0 software (SPSS Inc., Chicago, IL, USA). We considered *P*<0.05 to indicate statistical significance. The OR provides an effect estimate: a value of less than one assumes a protective effect, while a value of more than one assumes an increased disease risk. In addition, we analyzed the genotypic distributions of C4 SNPs in recipients with ABMR and stable recipients using logistic regression models adjusted for age, sex and immunosuppressive protocol.

## Abbreviations

ABMR: antibody-mediated rejection
HLA: human leukocyte antigens
MHC: major histocompatibility complex
DSA: donor-specific antibodies
HVEM: herpes virus entry mediator
BTLA: B and T lymphocyte attenuator
LIGHT: homologous to lymphotoxin, which exhibit inducible expression and compete with HSV glycoprotein D for binding to HVEM, a receptor expressed on T lymphocytes)
GC: germinal center
DC: dendritic cells
Tfh: helper cells
SNP: single nucleotide polymorphisms
HWE: Hardy-Weinberg equilibrium
NGS: next-generation sequencing
CRD: cysteine-rich domain
